# Hypoxia Negatively Regulates Antimetastatic PEDF in Melanoma Cells by a Hypoxia Inducible Factor-Independent, Autophagy Dependent Mechanism

**DOI:** 10.1371/journal.pone.0032989

**Published:** 2012-03-23

**Authors:** Asunción Fernández-Barral, José Luis Orgaz, Valentí Gomez, Luis del Peso, María José Calzada, Benilde Jiménez

**Affiliations:** 1 Department of Biochemistry, Universidad Autónoma de Madrid (UAM), Madrid, Spain; 2 Instituto de Investigaciones Biomédicas Alberto Sols, CSIC-UAM, Madrid, Spain; 3 Servicio de Inmunologia, Hospital de la Princesa, Instituto de Investigación Sanitaria Princesa and Departamento de Medicina, Universidad Autónoma de Madrid, Madrid, Spain; Enzo Life Sciences, Inc., United States of America

## Abstract

Pigment epithelium-derived factor (PEDF), a member of the serine protease inhibitor (*SERPIN*) superfamily, displays a potent antiangiogenic and antimetastatic activity in a broad range of tumor types. Melanocytes and low aggressive melanoma cells secrete high levels of PEDF, while its expression is lost in highly aggressive melanomas. PEDF efficiently abrogates a number of functional properties critical for the acquisition of metastatic ability by melanoma cells, such as neovascularization, proliferation, migration, invasiveness and extravasation. In this study, we identify hypoxia as a relevant negative regulator of PEDF in melanocytes and low aggressive melanoma cells. PEDF was regulated at the protein level. Importantly, although downregulation of PEDF was induced by inhibition of 2-oxoglutarate-dependent dioxygenases, it was independent of the hypoxia inducible factor (HIF), a key mediator of the adaptation to hypoxia. Decreased PEDF protein was not mediated by inhibition of translation through untranslated regions (UTRs) in melanoma cells. Degradation by metalloproteinases, implicated on PEDF degradation in retinal pigment epithelial cells, or by the proteasome, was also excluded as regulatory mechanism in melanoma cells. Instead, we found that degradation by autophagy was critical for PEDF downregulation under hypoxia in human melanoma cells. Our findings show that hypoxic conditions encountered during primary melanoma growth downregulate antiangiogenic and antimetastasic PEDF by a posttranslational mechanism involving degradation by autophagy and could therefore contribute to the acquisition of highly metastatic potential characteristic of aggressive melanoma cells.

## Introduction

Serine protease inhibitor (*SERPIN*) is a large superfamily of genes that codes for serine protease inhibitors in mammals [1]. However, there is a small number of SERPIN family members with non-inhibitory protease activity, among which is included pigment epithelium-derived factor (PEDF, gene symbol *SERPINF1*) [1]–[5].

PEDF was originally described as the most potent angiostatic factor in the eye [6]. PEDF is produced at high levels by retinal pigment epithelial (RPE) cells, and counteracts a number of potent angiogenic growth factors in the retina; ensuring the right balance of angiogenic regulators that leads to an optimum physiological pattern of blood vessels for a correct retinal function. A number of eye pathologies like diabetic retinopathy and eye-related macular degeneration are associated with loss of PEDF expression, leading to excessive and aberrant vascularization patterns associated with loss of vision [7].

Later studies showed that PEDF is also produced by a wide variety of epithelial cell types and its role in controlling primary tumor growth, angiogenesis and metastatic spread has been explored in a wide range of tumor types [8]–[10]. Levels of angiostatic PEDF decrease during the progression of a number of cancers, such as hepatocellular carcinoma [11], prostate carcinoma [12], [13], breast adenocarcinoma [14], glioblastoma [15] and Wilm's tumors [16].

We have recently shown that melanocytes are also among the cell types in our body that produce and secrete high levels of PEDF [17], which are comparable to the levels produced by RPE cells, neural cells or retinoblastoma cells. We [8], [17]–[19] and others [4], [20], [21] have described a complex mechanism underlying the potent inhibition of melanoma metastasis by PEDF. PEDF-mediated antitumor activity in melanoma and other tumors is based on its dual action on the tumor microenvironment and on the tumor cells themselves [8]. PEDF inhibits tumor angiogenesis by means of induction of apoptosis on endothelial cells and modulation of the angiogenic profile of melanoma cells. Additionally, PEDF exerts a potent inhibitory action on melanoma cells, inducing apoptosis under stress conditions (such as absence of growth factors or detachment from the extracellular matrix) and abrogating migration and invasion. More recently we have demonstrated that loss of PEDF expression enables melanoma cells to acquire migratory and invasive properties, as well as vasculogenic mimicry capability, which altogether is translated into an increased *in vivo* metastatic potential [17], [19]. Therefore, regulation of PEDF expression could be critical for the malignant progression of human melanoma.

The mechanisms responsible of PEDF reprogramming during the malignant progression of human melanoma are still elusive, and their identification could be of critical importance to better understand the biological significance of PEDF in melanoma. The skin is a mildly hypoxic microenvironment (pO_2_ in the dermal/epidermal junctions ranging from 0.5% to 10%) that significantly contributes to melanocyte transformation, as the result of hypoxia promoting both proliferation and survival, and avoiding senescence [22]. Thus, hypoxia has emerged as a relevant tumor-promoting environmental factor in skin melanocytes that cooperates with oncogenic BRAF (BRAF^V600E^) and activation of AKT pathway for malignant transformation [22]. Furthermore, hypoxia has been identified as a critical regulator of invasiveness and epithelial-mesenchymal transition (EMT) [23] thus promoting metastasis. Additionally, hypoxia is one of the main regulators of angiogenic growth factors and inhibitors, which contributes to tilt the balance toward inducers of angiogenesis and to impose the loss of relevant angiostatic factors during tumor progression [24].

Given the central role of hypoxia in tumor progression and angiogenesis, here we explored whether PEDF expression in human melanocytes and melanoma cell lines is regulated by variations in oxygen tension.

Cells respond to hypoxia through a combination of regulatory mechanisms that results in reduced oxygen consumption and restoration of oxygen supply. A central regulatory mechanism is based on modification of the gene expression profile mastered by the hypoxia-inducible factors (HIFs). HIF is a heterodimer comprising an oxygen-regulated alpha subunit (HIFα) and a constitutively expressed beta subunit (HIFβ). HIFα family comprises three members: HIF1α, HIF2α and HIF3α [25]–[27], which display differential expression and regulate the expression of a subset of non-overlapping target genes. Central to the hypoxia response is a family of 2-oxoglutarate dependent dioxygenases (EGL nine homolog, EGLNs; also called prolyl-hydroxylases, PHDs) that require oxygen as cosubstrate and constitute the main oxygen sensor mechanism so far characterized [28], [29]. PHDs hydroxylate HIFα in two proline residues [30], [31] and this posttranslational modification labels HIFα for proteasomal degradation. Reduced oxygen concentration in hypoxia comprises hydroxylation by PHDs and consequently HIFα subunits are stabilized. The stabilization of HIFα allows for the formation of the HIF1α/β heterodimer and lead to HIF-mediated transcription.

Transcriptional reprogramming through HIFs acts in concert with inhibition of translation through inactivation of the mammalian target of rapamycin (mTOR) and activation of the unfolded protein response (UPR); to effectively achieve hypoxia adaptation based on changes in metabolism, angiogenesis, endoplasmic reticulum (ER) homeostasis and autophagy [32], [33]. Hypoxia also regulates translation through miRNAs [34], [35] and regulation of RNA-binding proteins (RBPs) [36]. Additionally, selective degradation of certain target proteins under hypoxia by diverse degradation routes significantly contributes to hypoxia tolerance mechanisms [37], [38].

Here, we study the general characteristics of the mechanism responsible for regulation of PEDF expression by hypoxia in human melanocytes and melanoma cells. Our results show that reduction of PEDF production by hypoxia has common general characteristics with previously described regulation of PEDF in other cell types, and distinct characteristics that specifically involve degradation by autophagy in neural crest derived cells.

## Results

### Hypoxia Downregulates PEDF at the Protein Level in Melanocytes and Melanoma Cell Lines

**Figure 1:**
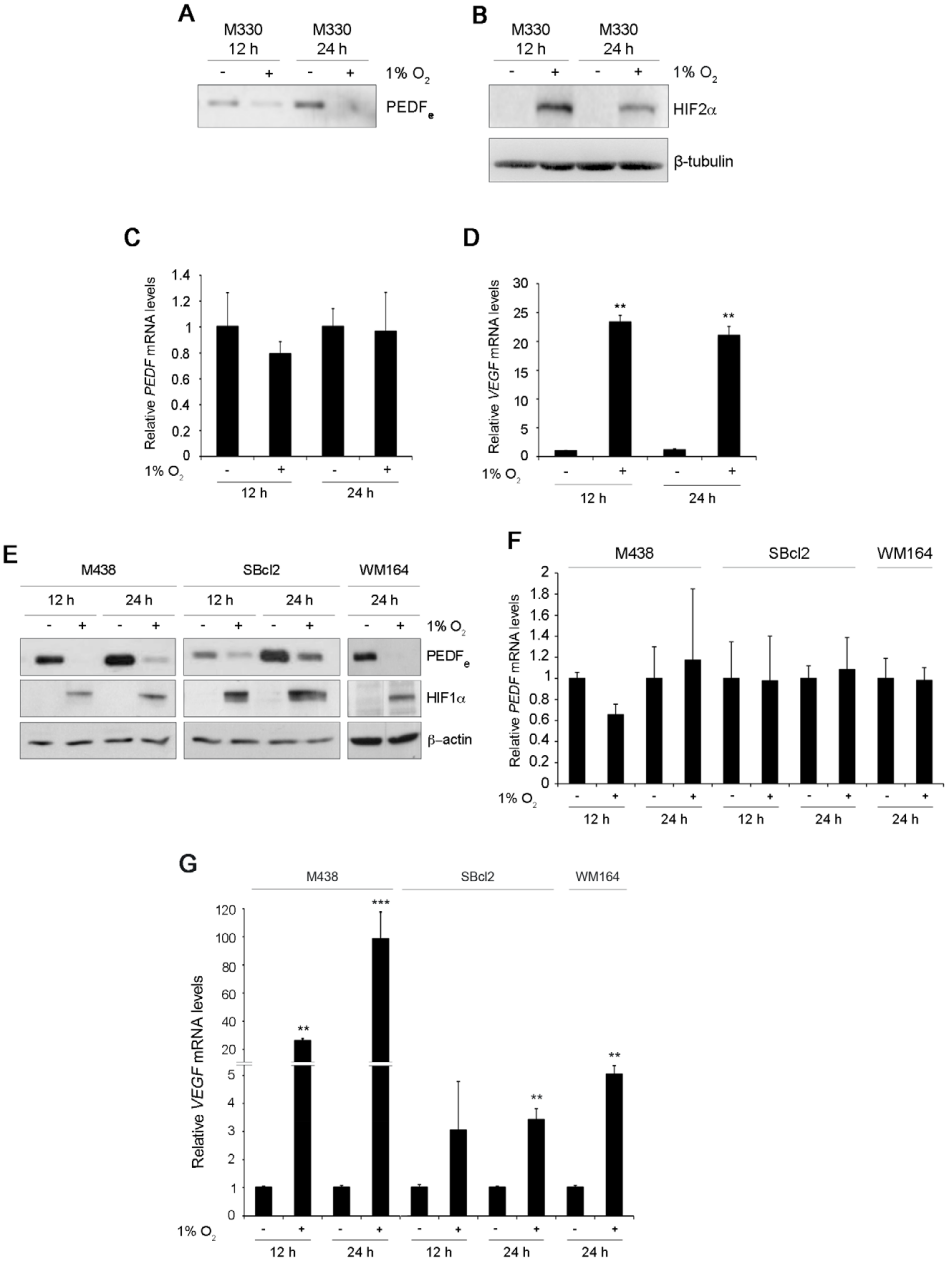
Hypoxia downregulates PEDF at the protein level in melanocytes and human melanoma cell lines. Western blot analysis of (A) extracellular PEDF (PEDF_e_) protein levels in conditioned medium (CM) and (B) HIF2α protein levels in whole-cell extracts (B) from M330 primary melanocytes incubated under normoxia (21% O_2_) or hypoxia (1% O_2_) for 12 h or 24 h. β-tubulin was used as loading control. Quantitative RT-PCR analysis of (C) *PEDF* mRNA levels and (D) *VEGF* mRNA levels in M330 primary melanocytes incubated in normoxia or hypoxia for 12 h or 24 h. *PEDF* and *VEGF* mRNA levels are shown relative to cells in normoxia after normalization to *β-actin*. Bars represent average ± standard deviation (SD) (**P<0.01). (E) Western blot analysis of PEDF_e_ protein levels in CM and HIF1α in whole-cell extracts from M438 primary melanocytes, SBcl2 and WM164 melanoma cell lines incubated in normoxia or hypoxia for 12 h or 24 h. β-actin was used as loading control. Quantitative RT-PCR analysis of (F) *PEDF* mRNA levels and (G) *VEGF* mRNA levels in M438 primary melanocytes, SBcl2 and WM164 melanoma cell lines incubated under normoxia or hypoxia for 12 h or 24 h. *PEDF* and *VEGF* mRNA levels are shown relative to normoxia after normalization to *β-actin*. Bars represent average ± SD (**P<0.01; ***P<0.001).

Seeking for regulators of PEDF relevant in the context of melanoma progression we explored whether hypoxia could be a candidate mechanism. In primary cultures of human skin melanocytes we found that extracellular levels of PEDF protein (PEDF_e_) detected by western blot analysis of conditioned medium gradually decreased under hypoxic (1% O_2_) (Fig. 1A) and anoxic (0% O_2_) conditions (Fig. S1). Downregulation of PEDF_e_ by hypoxia was detected at 8–12 h and secreted protein levels remained low after 24–48 h of hypoxia (Fig. 1A and data not shown). Establishment of hypoxia response in primary melanocytes was monitored by detection of hypoxia-inducible factor 2α (HIF2α) and 1α HIF1α stabilization by western-blot analysis of whole-cell extracts (Fig. 1B and data not shown). We next analyzed mRNA levels of PEDF in normoxic versus hypoxic conditions. Interestingly, we found that PEDF mRNA levels remained constant over the time course in which we detected downregulation of extracellular protein levels (Fig. 1C). VEGF mRNA levels were evaluated under the same experimental conditions as a well characterized HIF transcriptional target. As expected, hypoxia induced a large increase in VEGF mRNA levels in melanocytes (Fig. 1D). These results demonstrate that hypoxia downregulates secreted levels of PEDF at the protein level in melanocytes by posttranscriptional mechanisms.

Downregulation of extracellular PEDF by hypoxia was detected in serum-free conditioned medium and growth factor supplemented conditioned medium (Fig. S2A). Although PEDF is very efficiently secreted and consequently we detected low intracellular PEDF (PEDF_i_) levels in melanoma cell lines, additionally, we checked whether intracellular PEDF levels were modulated by hypoxia. Our results indicated that PEDF_i_ was downregulated by hypoxia and this downregulation was also independent of the presence or absence of growth factors (Fig. S2B). Rate of DNA synthesis was not affected by hypoxic conditions used in NHEM primary melanocytes and SBcl2 melanoma cell line (Fig. S3).

We also examined whether hypoxia downregulated PEDF in poorly aggressive melanoma cell lines that produce endogenous PEDF levels similar to primary melanocytes [17]. Hypoxia downregulated PEDF_e_ in SBcl2 and WM164 melanoma cell lines with a similar kinetics and extent to those found in primary melanocytes (the melanocyte primary culture M438 was used as a reference) (Fig. 1E). Also, in agreement with our results in primary melanocytes, PEDF mRNA levels were not modulated by hypoxia in the melanoma cell lines tested (Fig. 1F). Regulation of VEGF mRNA levels by hypoxia was used as positive control (Fig. 1G). VEGF mRNA induction by hypoxia was confirmed in primary melanocytes, SBcl2 and WM164 melanoma cell lines.

### Hypoxia Inducible Factor Does Not Mediate Downregulation of PEDF by Hypoxia in Melanocytes and Melanoma Cell Lines

**Figure 2:**
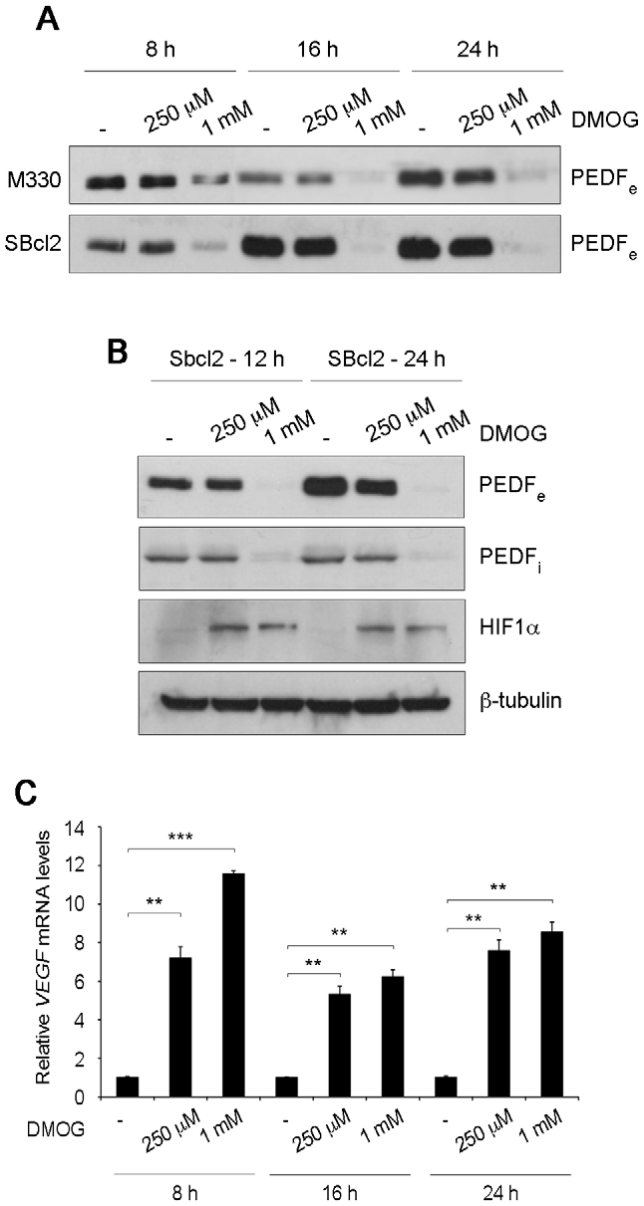
Inhibition of PHDs leads to decreased PEDF protein in normoxia in melanocytes and SBcl2 melanoma. (A) Western blot analysis of extracellular PEDF (PEDF_e_) protein levels in conditioned medium (CM) from M330 primary melanocytes (upper blot) and SBcl2 melanoma cell line (lower blot) treated with different concentrations of DMOG for 8 h, 16 h or 24 h. (B) Western blot analysis of PEDF_e_ protein levels in CM, intracellular PEDF (PEDF_i_) and HIF1α protein levels in whole-cell extracts from SBcl2 melanoma cells treated with DMOG for 12 h and 24 h. β-tubulin was used as loading control. (C) Quantitative RT-PCR analysis of *VEGF* mRNA levels in SBcl2 melanoma cell line treated with DMOG for the indicated times. *VEGF* levels are shown relative to controls without DMOG in time points, after normalization to *18s* rRNA. Bars represent average ± standard deviation (SD) (**P<0.01; ***P<0.001).

PHDs are the best characterized cellular oxygen sensors and they trigger many of the responses to hypoxia. Thus, we next decided to assess the effect of PHD inhibition on PEDF regulation. As PHDs require oxygen, iron ascorbate and 2-oxoglutarate as cosubstrates, we used the synthetic 2-oxoglutarate antagonist DMOG (N-(Methoxyoxoacetyl)-glycine methyl ester) to inhibit their activity. Treatment of primary melanocytes (M330) and SBcl2 melanoma cells with 1 mM DMOG led to a significant and time-dependent decrease in secreted PEDF levels detected by western blot of conditioned medium (Fig. 2A). When we analyzed the effect of DMOG in PEDF_i_ protein levels, we also found a similar dose response and kinetics as for the downregulation of extracellular PEDF by DMOG (Fig. 2B). As expected, we observed HIF1α stabilization after DMOG treatment (Fig. 2B), although a lower dose was required. The effects of DMOG were further confirmed analyzing the dose response and kinetics of VEGF mRNA levels (Fig. 2C). These results showed a significant difference between the DMOG dose required for maximum downregulation of PEDF protein levels and HIF stabilization; pointing that decreased PEDF protein was PHD-dependent but HIF-independent.

**Figure 3:**
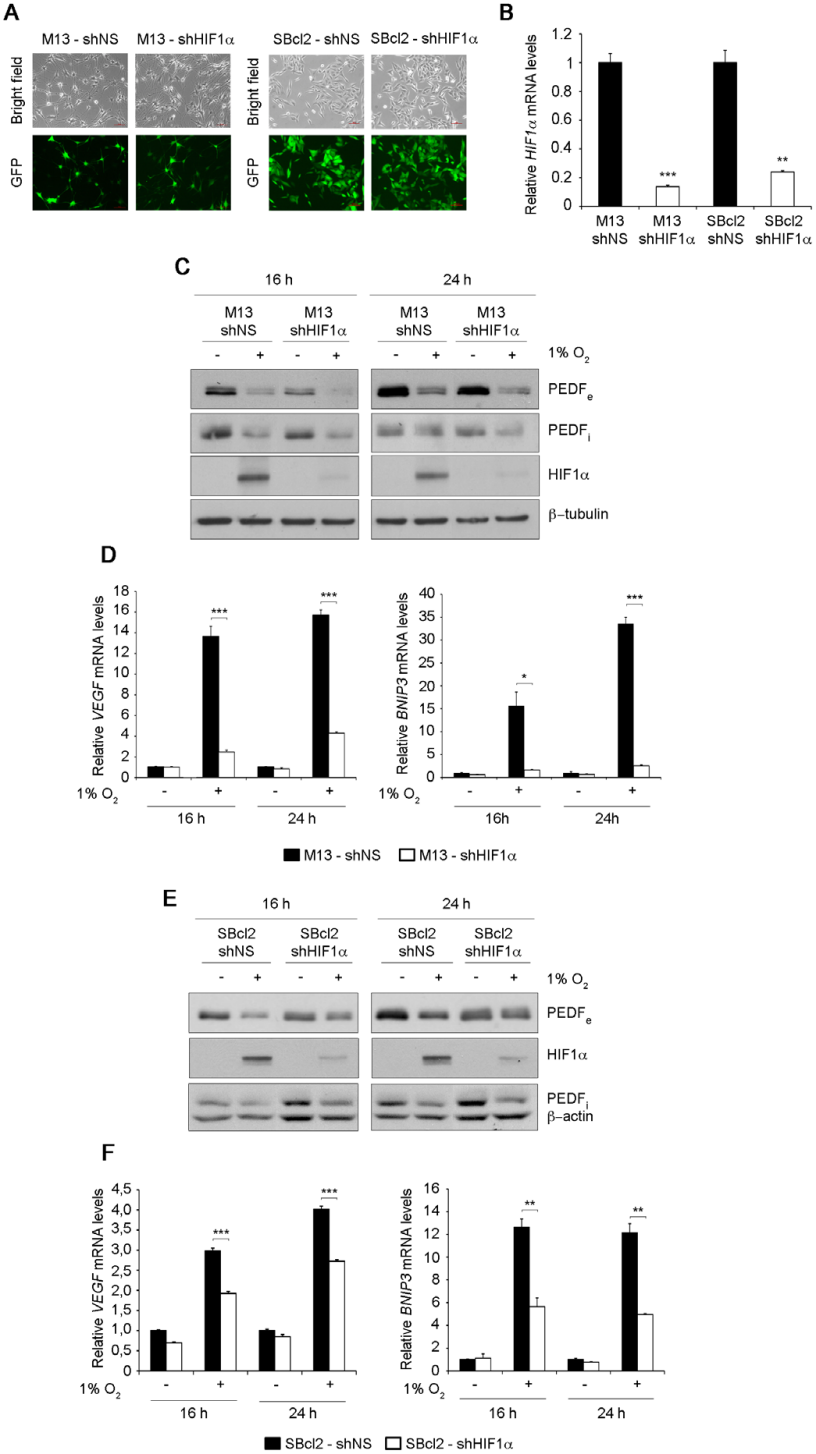
Hypoxia-induced downregulation of PEDF is HIF-independent in melanocytes and SBcl2 melanoma. (A) Transduction efficiency of M13 primary melanocytes (left panels) and SBcl2 melanoma cell line (right panels) after infection with non-silencing (shNS) or shRNA^mir^ to HIF1α (shHIF1α) lentivirus at multiplicity of infection of 40 (M13) or 60 (SBcl2). Fluorescence images (40× magnification) show more than 90% GFP-positive cells. (B) Quantitative RT-PCR analysis of *HIF1α* mRNA levels in M13-shNS, M13-shHIF1α primary melanocytes and SBcl2-shNS, SBcl2-shHIF1α melanoma cell lines. *HIF1α* mRNA levels are shown relative to control shNS cells after normalization to *18s* rRNA. Bars represent average ± standard deviation (SD) (**P<0.01; ***P<0.001). (C) Western blot analysis of extracellular PEDF (PEDF_e_) protein levels in conditioned medium (CM), intracellular PEDF (PEDF_i_) and HIF1α protein levels in whole-cell extracts from M13-shNS and M13-shHIF1α primary melanocytes incubated under normoxia (21% O_2_) or hypoxia (1% O_2_) for 16 h and 24 h. β-tubulin was used as loading control. (D) Quantitative RT-PCR analysis of *VEGF* (left panel) and *BNIP3* (right panel) mRNA levels in M13-shNS (filled bars) and M13-shHIF1α (empty bars) primary melanocytes. *VEGF* and *BNIP3* mRNA levels are shown relative to M13-shNS under normoxia after normalization to *18s* rRNA. Bars represent average ± SD (*P<0.05; ***P<0.001). (E) Western blot analysis of PEDF_e_ protein levels in CM, PEDF_i_ and HIF1α protein levels in whole-cell extracts from SBcl2-shNS and SBcl2-shHIF1α melanoma cell lines incubated under normoxia or hypoxia for 16 h and 24 h. β-actin was used as loading control. (F) Quantitative RT-PCR analysis of *VEGF* (left panel) and *BNIP3* (right panel) mRNA levels in SBcl2-shNS (filled bars) and SBcl2-shHIF1α (empty bars) melanoma cell lines. *VEGF* and *BNIP3* mRNA levels are shown relative to SBcl2-shNS under normoxia after normalization to *18s* rRNA. Bars represent average ± SD (**P<0.01; ***P<0.001).

To further explore whether HIF was involved in the decrease in PEDF protein levels imposed by hypoxia in primary melanocytes (M13) and SBcl2 melanoma cells, we silenced HIF1α expression using shRNA^mir^ to HIF1α (shHIF1α delivered by lentiviral transduction (Fig. 3). Non-silencing (NS) shRNA^mir^ (shNS) was used as control. Lentiviral transduction of primary melanocytes (M13) and SBcl2 cells was highly efficient as demonstrated by the high percentage of GFP positive cells (Fig. 3A). Efficiency of HIF1α silencing was determined analyzing HIF1α mRNA levels by quantitative RT-PCR, being this higher than 80% in primary melanocytes and SBcl2 melanoma cells (Fig. 3B). Despite the efficient silencing of HIF1α in primary melanocytes, hypoxic conditions reduced secreted and intracellular PEDF to a similar extent in shNS and shHIF1α cells (Fig. 3C). To further confirm HIF1α silencing in melanocytes we checked mRNA levels of the known HIF direct genes VEGF and BNIP3. Induction by hypoxia of both HIF genes in melanocytes was efficiently abrogated by shHIF1α (Fig. 3D). In agreement with our results in melanocytes, HIF1α silencing in SBcl2 melanoma cells did not interfere with downregulation of secreted PEDF protein levels by hypoxia (Fig. 3E). Interestingly, knock-down of HIF1α in SBcl2 melanoma cells diminished VEGF and BNIP3 mRNA induction by hypoxia, although to a lesser extent than observed in melanocytes (Fig. 3F). These results could be explained by differences in the mechanisms that mediate regulation of expression of HIF target genes in primary melanocytes versus melanoma cells.

### UTRs Are Not Required for PEDF Downregulation by Hypoxia in Melanoma Cell Lines

**Figure 4:**
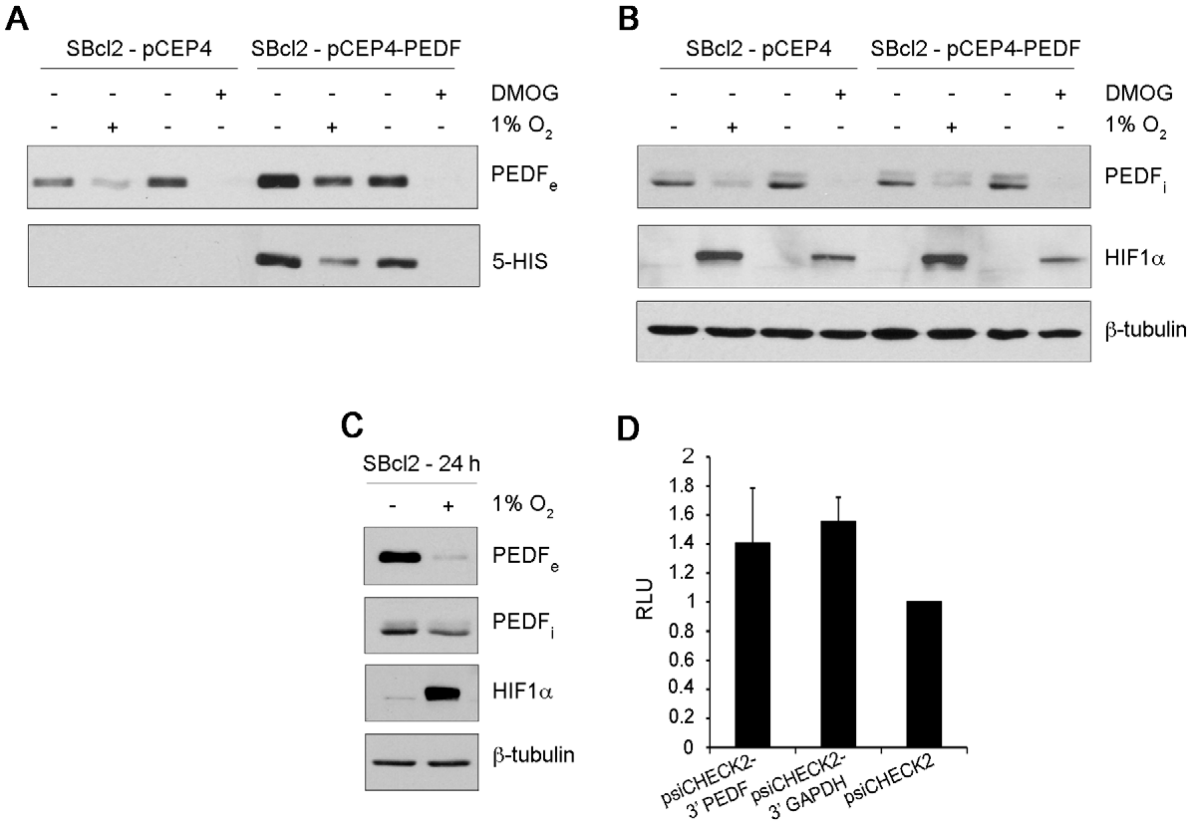
UTRs are not required for PEDF downregulation by hypoxia in SBcl2 melanoma. Western blot analysis from SBcl2-pCEP4 and SBcl2-pCEP4-PEDF melanoma cell lines incubated with 1 mM DMOG or under hypoxia (1% O_2_): (A) extracellular PEDF (PEDF_e_) and Penta-HIS (5-HIS) protein levels in 24 h conditioned medium (CM) and (B) intracellular PEDF (PEDF_i_) and HIF1α protein levels in whole-cells extract. β-tubulin was used as loading control. (C) Western blot analysis of PEDF_e_ protein levels in 24 h CM, PEDF_i_ and HIF1α protein levels in whole-cell extracts from SBcl2 melanoma cell line incubated under normoxia (21% O_2_) or hypoxia (1% O_2_). β-tubulin was used as loading control. (D) UTR-reporter assay in SBcl2 melanoma cell line transfected with a Renilla promoter reporter containing the 3′ UTR of PEDF (psiCHECK2-3′PEDF), the 3′UTR of GAPDH (psiCHECK2-3′GAPDH) or an empty reporter (psiCHECK2) and incubated under normoxia or hypoxia for 24 h. Renilla activity was normalized to luciferase activity, which is used as an internal control of transfection efficiency. psiCHECK2-3′GAPDH was used as a negative control. Bars represent average ± standard deviation (SD).

To further confirm that PEDF is regulated by hypoxia at the protein level in melanoma cells, we analyzed the effect of hypoxia on exogenous PEDF expressed from a heterologous promoter (CMV promoter in pCEP4-PEDF vector). SBcl2 melanoma cells were stably transfected with pCEP4 (SBcl2-pCEP4) or pCEP4-PEDF (SBcl2-pCEP4-PEDF). Downregulation of endogenous secreted PEDF by hypoxia and DMOG was confirmed in control SBcl2-pCEP4 cells (Fig. 4A). Regulation of exogenous PEDF protein was monitored by means of a histidine tag (5-HIS) fused to PEDF in pCEP4-PEDF vector. Figure 4A shows that hypoxia and DMOG efficiently reduced exogenous secreted PEDF protein levels in SBcl2-pCEP4-PEDF cells. Additionally, we confirmed that intracellular PEDF protein levels decreased in SBcl2-pCEP4 and SBcl2-pCEP4-PEDF cells under hypoxia or DMOG treatment (Fig. 4B). Stabilization of HIF1α protein as a control of hypoxia response is shown in all the experimental conditions used (Fig. 4B). Given that exogenous PEDF expressed from pCEP4-PEDF vector lacked the 5′ and 3′ untranslated regions (UTRs), downregulation of PEDF by hypoxia was most likely not mediated through UTR inhibition of translation. In order to directly asses the role of SERPINF1 UTRs in the regulation of PEDF by hypoxia in melanoma cells we cloned the 3′UTR of PEDF in a Renilla reporter construct psiCHECK2 (psiCHECK2-3′PEDF) and analyzed the 3′UTR of PEDF using UTR reporter assays in normoxic versus hypoxic conditions in melanoma cells. We used the 3′UTR of GAPDH (psiCHECK2-3′GAPDH) as a control, together with the empty psiCHECK2 plasmid, since it was previously shown that hypoxia did not modify the translation of GAPDH mRNA [39]. We first confirmed that hypoxia downregulated extracellular and intracellular PEDF protein levels (Fig. 4C) in SBcl2 cells. As in previous experiments hypoxia response in SBcl2 cells was confirmed by stabilization of HIF1α detected by western blot analysis of whole-cell extracts (Fig. 4C). Afterward, in transient transfection experiments, we found no significant differences in reporter activity when we compared psiCHECK2-3′UTR PEDF with empty vector or psiCHECK2-3′UTR GAPDH (Fig. 4D), indicating that the 3′UTR of PEDF does not mediate inhibition of translation under hypoxic conditions. These results were also confirmed in M000921 human melanoma cell line in which PEDF (intracellular and extracellular) is highly regulated by hypoxia and presents a higher efficiency of transfection (Fig. S4).

In summary, these results support that PEDF downregulation is not mediated by regulation of translation through UTRs.

### Degradation by Metalloproteinases or the Proteasome Does Not Mediate Downregulation of PEDF by Hypoxia in Melanoma Cell Lines

As no difference in PEDF mRNA level was detected in hypoxia compared to normoxia in melanocytes or melanoma cells, and inhibition of translation through UTRs was not involved in the downregulation of PEDF, it stands to reason that hypoxic regulation of PEDF could likely occur at the posttranslational level.

**Figure 5:**
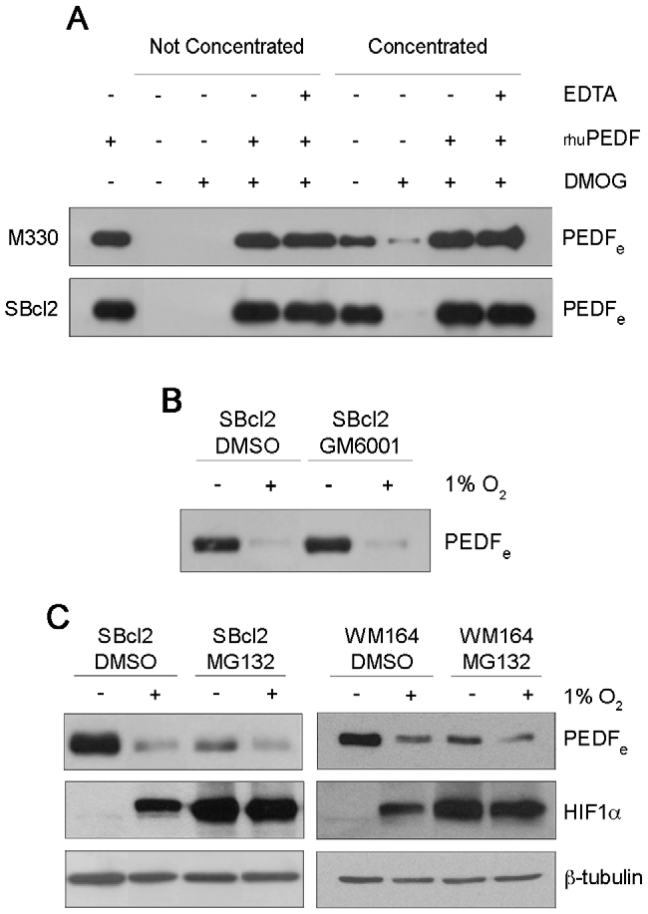
Hypoxia-induced downregulation of PEDF in melanocytes and SBcl2 melanoma cells is not mediated by metalloproteinases or proteasomal degradation. (A) Western blot analysis of extracellular PEDF (PEDF_e_) protein levels in 24 h conditioned medium (CM) from M330 primary melanocytes (upper blot) and SBcl2 melanoma cell line (lower blot). Cells were treated with 1 mM DMOG for 24 h and the CM were incubated with 100 ng human recombinant PEDF (rhuPEDF) and 20 mM EDTA at 37°C for 2 h. (B) Western blot analysis of PEDF_e_ protein levels in CM from SBcl2 melanoma cell line treated with metalloproteinase inhibitor GM6001 (10 µM) and incubated under normoxia (21% O_2_) or hypoxia (1% O_2_) for 24 h. (C) Western blot analysis of PEDF_e_ protein levels in 16 h CM and HIF1α protein levels in whole-cell extracts from SBcl2 and WM164 melanoma cell lines after treatment with the proteasome inhibitor MG132 (5 µM and 1 µM respectively) under normoxia or hypoxia. β-tubulin was used as loading control.

Matrix metalloproteinases type 2 (MMP-2) and type 9 (MMP-9) belong to the large MMP family of Zn^2+^- and Ca^2+^-dependent extracellular proteinases that are critically involved in the regulation of migration, invasion and angiogenesis [40], [41]. It has been shown that PEDF produced by RPE cells is degraded extracellularly by MMP-2 and MMP-9 activated in hypoxic conditions [37]. Therefore, we studied whether this posttranslational regulatory mechanism could be responsible of decreased the PEDF protein in neural crest-derived pigment cells (melanocytes and melanoma cells) under hypoxia. Conditioned medium from control or DMOG-treated melanocytes and SBcl2 cells were tested for protein degradation activity against exogenously added purified recombinant human PEDF (rhuPEDF). *In vitro* incubation of rhuPEDF with either direct or concentrated conditioned medium from DMOG-treated melanocytes and SBcl2 cells did not produce any significant degradation of the exogenous PEDF protein (Fig. 5A). Incubation with EDTA was used to inhibit expected induction of metalloproteinase activity by hypoxia. Downregulation of endogenous secreted PEDF protein by hypoxia was confirmed in concentrated conditioned medium from control versus DMOG-treated melanocytes and SBcl2 cells (Fig. 5A). Furthermore and in agreement with previous results, cells treated with the metalloproteinase inhibitor GM6001 did not block downregulation of secreted PEDF protein levels by hypoxia in SBcl2 melanoma cells (Fig. 5B).

We next studied whether decreased PEDF protein levels under hypoxia were a consequence of degradation by the proteasome, and found that the diminished PEDF_e_ protein levels in hypoxia were not recovered when we treated SBcl2 and WM164 cells with the proteasome inhibitor MG132 (Fig. 5C). As expected, MG132 stabilized HIF1α in normoxic conditions (Fig. 5C).

Hence, these results indicated that neither extracellular degradation by metalloproteinases nor proteasomal degradation was implicated in the downregulation of PEDF protein levels by hypoxia in melanocytes and melanoma cells.

### Downregulation of PEDF by Hypoxia in Melanoma Cell Lines Involves Degradation by Autophagy

**Figure 6:**
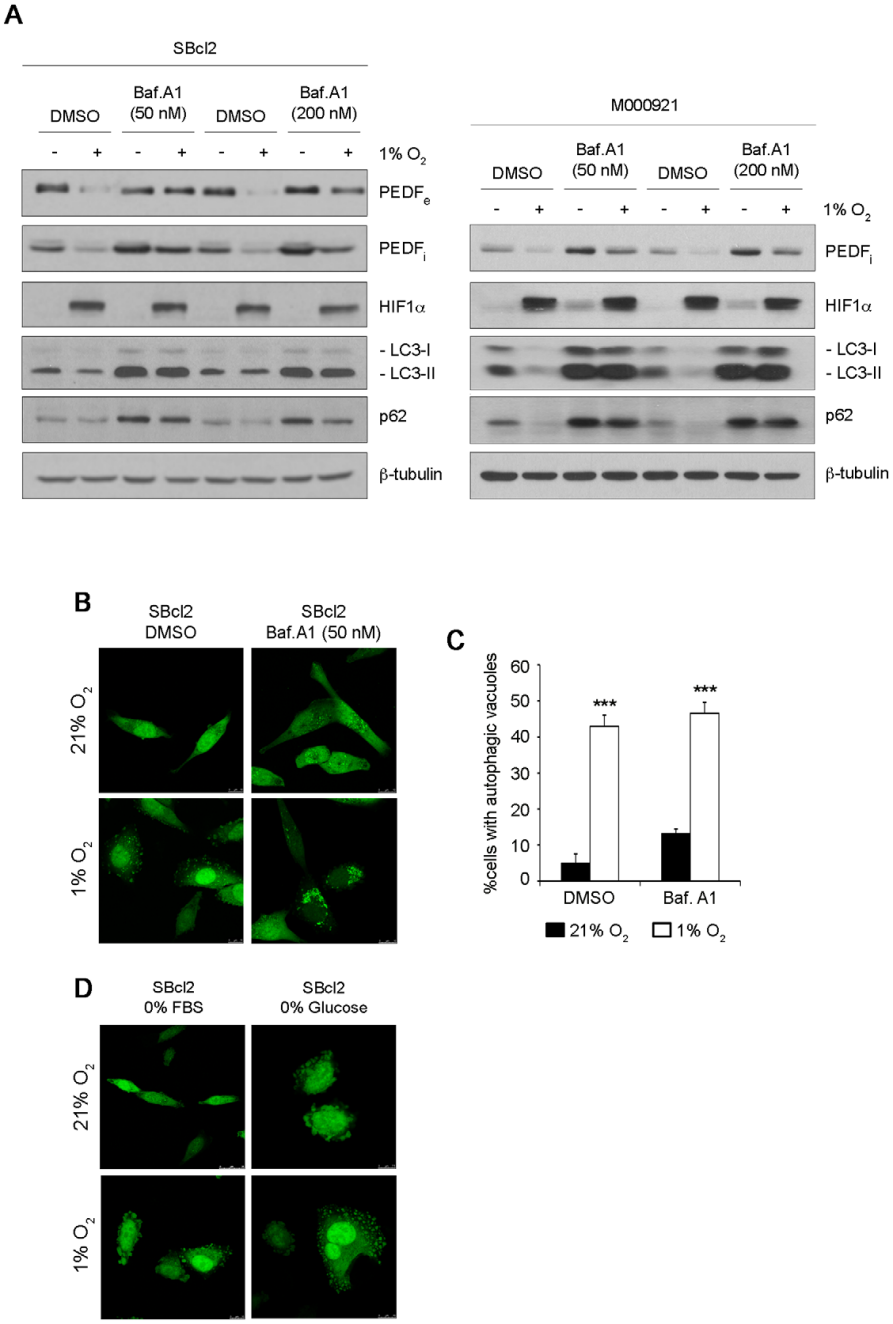
Autophagy is involved in downregulation of PEDF by hypoxia in melanoma cells. (A) Western blot analysis of extracellular PEDF (PEDF_e_) protein levels in 24 h conditioned medium (CM), intracellular PEDF (PEDF_i_), HIF1α LC3 and p62 protein levels in whole-cell extracts from SBcl2 (left) and M000921 (right) melanoma cell lines treated with different concentrations of the autophagy inhibitor bafilomycin A1 (Baf. A1, 50 nM and 200 nM) or DMSO vehicle under normoxic (21% O_2_) or hypoxic (1% O_2_) conditions. β-tubulin was used as loading control. (B) Fluorescence images (63× magnification) of GFP-LC3 protein redistribution in SBcl2 melanoma cell line (transduced with pLV-EGFP-LC3 plasmid) treated with 50 nM Baf. A1 in normoxia or hypoxia for 24 h. (C) Quantification of SBcl2 cells with autophagic vacuoles after Baf. A1 treatment for 24 h under normoxia (filled bars) or hypoxia (empty bars). Ten fields from each condition were counted for quantification. Bars represent average ± standard deviation (SD) (***P<0.001). (D) Fluorescence images (63× magnification) of GFP-LC3 redistribution in SBcl2 melanoma cell line grown in the absence of growth factors (0% FBS) or ischemic conditions (0% glucose) under normoxia or hypoxia.

Autophagy is a tightly controlled degradation pathway that has been recently shown to be activated in response to hypoxia [42]–[44]. Among the identified autophagy-related proteins, microtubule-associated protein light chain 3 (LC3) has been widely used to monitor the autophagic response [45], [46]. LC3 exists in two forms: LC3-I (18 kDa) localized in the cytosol and its proteolytic derivative LC3-II (16 kDa) which is modified by conjugation with phosphatidylethanolamine and bound to autophagosomal membranes. Additionally, decreased levels of sequestosome 1 (SQSTM1/p62) were used to monitor induction of autophagy by hypoxia [44]. Hypoxia induced accumulation of LC3-II positive autophagic vacuoles in SBcl2 cells with a rapid and sustained kinetics (Fig. S5). Induction of autophagy by hypoxia was further demonstrated by decreased the levels of LC3-II and p62 in SBcl2 and M000921 melanoma cells (Fig. 6A). Quantification of SBcl2 cells with autophagic vacuoles showed a significant increase (***P<0.001) in hypoxia versus normoxia (Fig. 6C). As an additional control we show that ischemic conditions mimicked by glucose starvation induced a similar pattern of accumulation and distribution of GFP-LC3 signal than hypoxia in SBcl2 cells (Fig. 6D).

To investigate whether autophagy was implicated on the degradation of PEDF by hypoxia in SBcl2 melanoma cells we used bafilomycin A1 (Baf. A1), which inhibits the vacuolar ATPase and blocks the fusion of autophagosomes with lysosomes [47]. As expected, Baf. A1 treatment of SBcl2 and M000921 cells induced accumulation of LC3-II and p62 detected by western-blot (Fig. 6A) and redistribution of GFP-LC3 fusion construct into punctuate cytoplasmic structures indicative of accumulation of autophagic vacuoles (Fig. 6B, C). Figure 6A shows that Baf. A1 treatment efficiently blocked downregulation of PEDF_e_ and PEDF_i_ (Fig. 6A) by hypoxia.

**Figure 7:**
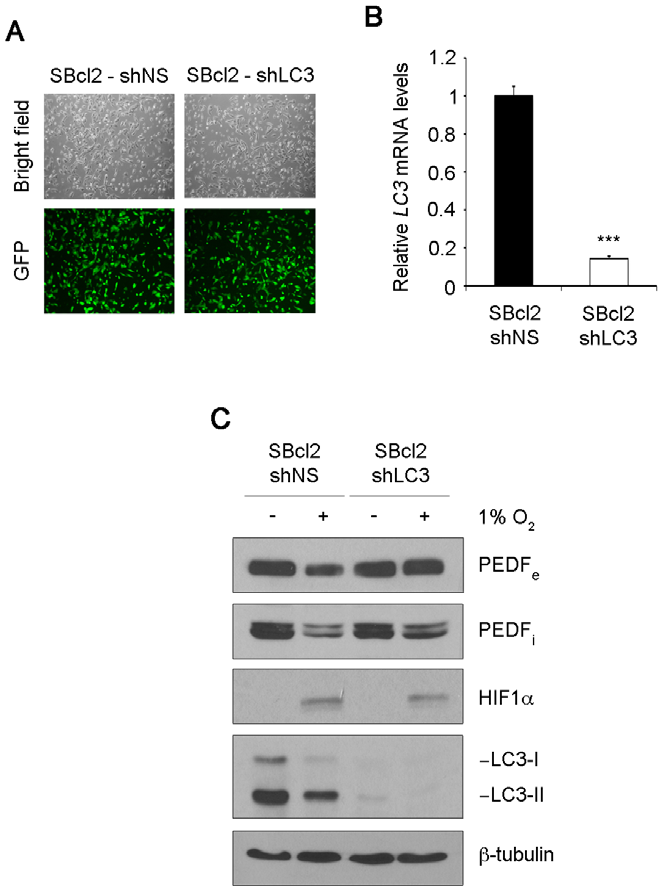
LC3 knock-down prevents downregulation of PEDF by hypoxia in melanoma cells. (A) Transduction efficiency of SBcl2 melanoma cell line after infection with non-silencing (shNS) or shRNA^mir^ to LC3 (shLC3) lentivirus at multiplicity of infection of 60. Fluorescence images (20× magnification) show more than 90% GFP-positive cells. (B) Quantitative RT-PCR analysis of *LC3* mRNA levels in SBcl2-shNS and SBcl2-shLC3 melanoma cell lines. *LC3* mRNA levels are shown relative to SBcl2-shNS after normalization to *18s rRNA*. Bars represent average ± standard deviation (SD) (***P<0.001). (C) Western blot analysis of extracellular PEDF (PEDF_e_) protein levels in conditioned medium (CM), intracellular PEDF (PEDF_i_), HIF1α and LC3 protein levels in whole-cell extracts from SBcl2-shNS and SBcl2-shLC3 melanoma cells incubated under normoxia (21% O_2_) or hypoxia (1% O_2_) for 24 h. β-tubulin was used as loading control.

Implication of autophagy in the degradation of PEDF under hypoxia was further confirmed by silencing of LC3 in SBcl2 melanoma cells. LC3 was efficiently interfered in SBcl2 melanoma cells using shRNA^mir^ to LC3 (Fig. 7A–B). Silencing of LC3 prevented PEDF donwregulation by hypoxia (Fig. 7C).

These results implied that autophagy-mediated degradation of PEDF is induced in response to hypoxia in melanoma cells.

## Discussion

PEDF was first identified as an endogenous inhibitor of angiogenesis in the eye [48]. RPE cells secrete high levels of PEDF to ensure a proper balance of neovascularization in the retina. Avascular eye compartments like the cornea and the vitreous are rich in PEDF. Therefore, PEDF plays a pivotal role on maintaining the eye vasculature in a quiescent state and loss of its expression is associated with pathological neovascularization leading to compromised vision and blindness [6].

We have recently shown that PEDF is also produced at high levels by neural crest-derived pigment-producing cells, the skin melanocytes [17]. The role of PEDF in the control of physiological skin vascularization remains to be characterized. Given PEDF's role as an antiangiogenic factor, we have recently described the regulation of its expression during the malignant progression of human melanomas and the functional consequences of loss of PEDF expression [17]. PEDF expression is high in melanoctyes, but it is lost during malignization of human melanoma. *In vitro* and *in vivo* functional analysis combined with interference strategies to silence PEDF, led us to demonstrate that PEDF has a broad function in melanoma that allows it to dually impinge on the vascular component of the tumor microenvironment and on directly counteracting a set of capabilities that enable the metastatic spread of melanoma cells [17], [19].

The functional relevance and multifunctionality of PEDF in melanoma prompted us to identify an important regulatory mechanism during melanoma progression. Our results suggest that PEDF expression could be modulated by two general types of mechanisms, reprogramming events and loss of expression [17].

Hypoxia is a hallmark of tumors that results from an imbalance between oxygen supply and consumption in continuously proliferating cancer cells in a tumor mass devoid of an adequate vascular network to cope with imposed oxygen demand. Consequently, hypoxia is one of the main triggers of tumor neovascularization and has been shown to contribute to tumor cell invasion, migration and metastasis [49].

Signals from the microenvironment such as hypoxia and inflammation are thought to reprogram and switch melanoma cells toward an invasive phenotype [50], and therefore, could be responsible for the reprogramming of PEDF during melanoma progression.

Previous reports described that PEDF is decreased by hypoxia in retinoblastoma [48] and RPE cells [37], although none of them studied the role of HIF in PEDF downregulation. Here, we describe the general characteristics of the mechanism responsible of decreased PEDF protein levels under hypoxia in human melanocytes and melanoma cells. We show that secreted PEDF, as well as intracellular PEDF protein levels, decrease under low oxygen conditions in primary melanocytes and several human melanoma cell lines. However, we found no significant differences in PEDF mRNA levels when we compared hypoxic versus normoxic conditions, suggesting that regulation of PEDF by hypoxia was posttranscriptional. This result is in agreement with previous studies in retinoblastoma cells and RPE cells in which diminished PEDF protein levels under hypoxia did not correlate with changes in PEDF mRNA levels [37], [48].

Moreover, in support to our results, the meta-analysis of gene profiling data sets from 16 independent experiments by Ortiz-Barahona and collaborators [51] confirmed there was no variation in PEDF mRNA levels in normoxic versus hypoxic conditions (data not shown).

HIF plays a central role in the regulation of the cell responses that allows adaptation to reduced oxygen tension. Although PEDF was not regulated at the transcriptional level, molecules downstream HIF could be involved in PEDF downregulation. We therefore analyzed whether HIF was implicated in the observed effects on PEDF in primary melanocytes and melanoma cell lines. Using lentiviral transduction of shRNA specific to HIF1α, we demonstrated that decreased PEDF protein levels in melanocytes and melanoma cells were not mediated by HIF1α.

Alternative mechanisms that participate in the downregulation of specific targets under hypoxia are the following: (i) regulation of translation by miRNAs and RBPs [52], (ii) selective degradation of hypoxia targets by the proteasome [38] or metalloproteinases [37] (iii) inhibition of translation through mammalian target of rapamycin (mTOR) kinase, (iv) activation of the unfolded protein response [33], [53] and (v) degradation by autophagy [42]–[44].

Inhibition of overall protein synthesis has long been accepted as a general trait of adaptation to hypoxia [54]. Notwithstanding, translation of specific mRNAs is favored by low oxygen tension. This is the case of the mRNAs of *HIF1α* [39] and *VEGFA* [55] which harbor regulatory elements that promote their preferential translation under overall translation inhibition imposed by hypoxia. We have directly addressed whether downregulation of PEDF by hypoxia occurs at the translational or posttranslational level. Two independent approaches allowed us to conclude that translation of PEDF mRNA is unlikely to be regulated by low oxygen tension. First, exogenous PEDF produced using vector constructs lacking the 5′UTR and 3′UTR was effectively decreased under hypoxic conditions. Furthermore a reporter construct of PEDF 3′UTR was not affected by hypoxic conditions in several melanoma cell lines. However, the UTRs are highly conserved in *SERPINF1* among different species, which points to their putative regulatory role by an as yet unidentified mechanism. Secondly, we used a bioinformatic approach to predict putative targets sequences for the RBPs HuR and TIA-1 [56], [57]. This program identifies motifs that share two common characteristics: (i) a primary sequence over 20 bp rich in AU and (ii) a specific secondary structure named stem-loop. With this approach we identified a small region rich in AU in the 3′ UTR of *SERPINF1* (data not shown). However, this sequence was in a non-conserved region and it was not identified by the *in silico* analysis as a putative RBP binding site; most likely due to lack of the secondary structure necessary for the binding of HuR or TIA-1.

These results prompted us to directly address the implication of degradation pathways relevant in the context of hypoxia response as the main mechanism underlying decreased PEDF protein levels under low oxygen tension conditions in melanocytes and melanoma cells.

The first candidate that we explored was the proteasome. The proteasome has been recently implicated on the selective degradation of specific targets under low oxygen conditions. This mechanism is responsible of downregulating the ternary complex factor net under hypoxia [38]. However, inhibition of the proteasome using MG132 did not block downregulation of PEDF levels by hypoxic conditions in melanoma cell lines. Also, the fact that decreased PEDF protein levels under low oxygen tension were not mediated by the proteasome makes it unlikely that activation of the UPR could be involved.

Taking into account a previously reported implication of metalloproteinases in the downregulation of PEDF protein levels under hypoxia in RPE cells [37] we checked whether this mechanism could be also operating in neural crest-derived pigment-producing cells. Our results demonstrate that induction of metalloproteinases by hypoxia was not responsible of decreased PEDF protein levels in melanoma cell lines.

An alternative degradation pathway that has been recently demonstrated to be relevant to the hypoxia adaptation response is degradation by autophagy [33], [42]–[44]. Specific targets downregulated by hypoxia have been shown to be degraded by autophagy. We show that hypoxic conditions significantly induced autophagy in melanoma cells as revealed by the punctuated phenotype of GFP-LC3 labeling and downregulation of LC3-II and p62 levels. The autophagy inhibitor Bafilomycin A1 (Baf. A1), which blocks the fusion of autophagosomes with lysosomes, abrogated downregulation of PEDF protein levels under low oxygen tension in melanoma cells. Silencing of LC3 also prevented PEDF downregulation by hypoxia in melanoma cells. These results support that decreased extracellular PEDF levels by hypoxia are a consequence of degradation by autophagy of intracellular PEDF, resulting in loss of its biological activities in pigment-producing melanocytes and melanoma cells.

Altogether our results point to hypoxia as a permissive environment associated with decreased production of PEDF by melanocytes and melanoma cells that in turn impacts on the acquisition of a more malignant phenotype. Furthermore, downregulation of PEDF at low oxygen tension is HIF-independent and occurs at the level of protein degradation involving the participation of autophagy as the most likely candidate mechanism. Both hypoxia and autophagy play a significant role in the context of melanoma progression [22], [58]–[60], therefore we have identified a relevant mechanism that may underlie reprogramming of PEDF expression during the malignant progression of melanoma. Loss of PEDF expression during melanoma malignization enables acquisition of angiogenic, invasive and metastatic capabilities to melanoma cells [17], [19]. Hypoxic conditions at the invasive front could be responsible of required decreased PEDF levels to enable low proliferation and increased migration and invasiveness characteristic of invasive phenotype melanoma cells. Both heterogeneity in tumor oxygenation, as well as colonization of new tissue environments characterized by higher oxygen tensions than the skin may lead to PEDF regulation in melanoma lesions [17] and subsequent reprogramming back to high PEDF to allow melanoma cells to gain the proliferative potential required to successfully colonize target organs.

## Materials and Methods

### Ethics statement

The present study was approved by the institutional Review Board of Children's Hospital Universitario Niño Jesús (Madrid, Spain) in accordance with the Helsinki Declaration. Informed written consent was obtained from all donors of foreskins.

### Cell Culture

Human melanoma cell lines SBcl2 (radial growth phase) and WM164 (established from a metastasis) were provided by Dr Herlyn (The Wistar Institute, Philadelphia, PA, USA) and cultured as described previously [61]. M000921 melanoma cell line (established from a metastasis) was provided by Dr Hoek (University Hospital of Zürich, Zürich, Switzerland) and cultured as reported earlier [62]. Primary human melanocytes were isolated from foreskins from independent donors and grown as described previously [63]. Primary cultures of melanocytes obtained from different single donors were used in this study (M330, M438, M13). For experiment in Figure S3, primary human melanocytes from Lonza (Basel, Switzerland) obtained from single donors were also used (NHEM). Primary cultures of melanocytes were used between passages 4–6, thus, a limited number of experiments could be carried out for each particular primary culture and therefore three different cultures were used.

### Reagents and Antibodies

The prolyl-4-hydroxylase inhibitor DMOG (N-(Methoxyoxoacetyl)-glycine methyl ester, 250 µM and 1 mM) was purchased from Enzo Life Sciences (Farmingdale, NY, USA); EDTA (20 mM) and autophagy inhibitor Bafilomicyn A1 (Baf.A1) (50 nM and 200 nM) were purchased from Sigma (St Louis, MO, USA). The metalloproteinase inhibitor GM6001 (10 µM) and the proteasome inhibitor MG132 (1 µM and 5 µM) were from Calbiochem (Darmstadt, Germany). Antibodies used were: extracellular PEDF (polyclonal; Bioproducts, West Palm Beach, FL, USA), intracellular PEDF (monoclonal; Chemicon, Billerica, MA, USA), HIF1α (monoclonal; BD Transduction Laboratories, Franklin Lakes, NJ, USA), HIF2α polyclonal; Abcam, Cambridge, UK), β-tubulin (monoclonal; Sigma), β-actin (polyclonal; Santa Cruz Biotechnologies, Santa Cruz, CA, USA), p62 (polyclonal; Cell Signaling Technology, Danvers, MA, USA), LC3 (polyclonal; Cell Signaling Technology) and Penta-His (monoclonal; Qiagen, Hilden, Germany).

### Hypoxia

Hypoxia conditions were achieved by incubating cells in a Hypoxystation H35 (Don Whitley Scientific Limited, Shipley, UK). Hypoxia was generated using a gas mixture of 1% O_2_, 5% CO_2_ and 94% N_2_. In Figure S4 anoxia was generated using a gas mixture of 0% O_2_, 5% CO_2_ and 95% N_2_. Alternatively, hypoxic like responses were mimicked by incubation with 250 µM and 1 mM DMOG (Enzo Life Sciences).

### Conditioned Medium Preparation

For extracellular PEDF (PEDF_e_) detection, cells were cultured in basal medium. Later, conditioned medium (CM) was collected, centrifuged to eliminate cellular debris and treated with protease inhibitor PMSF (phenylmethylsulfonyl fluoride; Sigma). CM was concentrated 50 times using Amicon Ultra (Millipore, Billerica, MA, USA) devices with 10 kDa cut-off in a refrigerated centrifuge. Afterward, 15–30 µl of CM was loaded per lane and used for western blot analysis.

### Metalloproteinase Assay

A total of 100 ng recombinant human PEDF (rhuPEDF) was mixed in 10 µl of SBcl2 direct or concentrated CM and when indicated was treated with EDTA (Sigma) protease inhibitor at 37°C for 1 h. Reaction mixture was subjected to western blot for PEDF_e_ detection.

### Western blotting

Whole-cell extracts were prepared by lysing the cells in Laemmli buffer (50 mM Tris-HCl pH 6.8, 2% SDS, 10% glycerol, 0.1% bromophenol blue and 100 mM DTT) containing protease and phosphatase inhibitors (10 µg/ml leupeptin; 10 µg/ml aprotinin; 10 µg/ml sodium orthovanadate; 1 µM PMSF (all from Sigma)). Whole-cell extracts or CM were separated by SDS-PAGE and subsequently transferred to PVDF membranes, and afterward, incubated with appropriate primary and horseradish-conjugated secondary antibodies and developed with ECL (GE Healthcare, Buckinghamshire, UK). Shown data are from a representative experiment that was confirmed on at least two independent experiments.

### EdU assay

DNA synthesis in primary human melanocytes (NHEM) and SBcl2 melanoma cell lines in normoxic vs hypoxic conditions was analyzed by the incorporation of 5-ethynyl-2-deoxyuridine (EdU) using Click-iT EdU Imaging Kit (Invitrogen, Paisley, UK) as indicated by the manufacturer. EdU-positive nuclei were counted in six independent fields using a TCS SP5 DM16000 spectral confocal microscope (Leica Microsystems, Heidelberg, Germany). Nuclei were visualized using DAPI (4′,6-diamidino-2-phenylindole).

### RNA Extraction and Quantitative RT-PCR

Total RNA was extracted and purified with the RNeasy Mini Kit (Qiagen). Total RNA (1 µg/sample) was reverse transcribed to cDNA (Improm-II reverse transcriptase; Promega, Madison, WI, USA) and 1 µl of cDNA samples were used as template for amplification reactions carried out with the LC Fast Start DNA master SYBR Green I Kit (Roche Applied Science, Basel, Switzerland) following the manufacturer's instructions. Oligonucleotides used were: 28s rRNA sense 5′-cagtacgaatacagaccg-3′ and antisense 5′-ggcaacaacacatcatcag-3′; β-actin sense 5′-cccagagcaagagagg- 3′ and antisense 5′-gtccagacgcaggatg-3′; HIF1α sense 5′-gtttactaaggacaagtcacc-3′ and ansitense 5′-ttctgtttgttgaagggag-3′; and BNIP3 sense 5′-gtctggacggagtagc-3′ and antisense 5′-ggccgacttgaccaat-3′. For detection of mRNA levels of 18s rRNA, PEDF, VEGF and LC3B, 1 µl of cDNA samples were used as a template and amplified with ABI Prism 7900 HT (Applied Biosystems, Carlsbad, CA, USA) using the following TaqMan probes (Applied Biosystems): 18s rRNA (Hs99999901_s1), PEDF (Hs00171467_m1), LC3B (Hs00797944_s1) and VEGF (Hs00173626_m1). Average ± standard deviation (SD). Values shown are from a representative experiment that was confirmed on at least three independent experiments.

### Lentivirus Production and Transduction of Target Cell Lines

Lentiviruses were produced and tittered as previously reported [64]. For transduction of cell lines, lentiviruses were used at multiplicity of infection (MOI) of 40–60 in the presence of 8 µg/ml polybrene (Sigma) for 8 h. Transduction efficiency was higher than 90%, and after 72 h, gene overexpression or knock-down was assessed.

### LC3 Overexpression

For LC3 overexpression we used the lentiviral vector pLV-EGFP-LC3 plasmid, kindly provided by Dr Soengas (Centro Nacional de Investigaciones Oncologicas, Madrid, Spain).

### RNA Interference

HIF1α and LC3 silencing was carried out using the lentiviral vector pGIPz containing shRNA^mir^ sequence V2LHS_132150 and V3LHS_408637 respectively from Open Biosystems (Thermo Fisher Scientific, Huntsville, AL, USA). Non-silencing shRNA^mir^ sequence (shNS), with no homology to known mammalian genes was used as control, cloned in a pGIPz vector (Open Biosystems).

### Generation of PEDF-overexpressing Cell Lines

SBcl2 and M000921 melanoma cell lines were seeded on 21 cm^2^ plates (10^6^ cells) and 24 h later were transfected using Lipofectamine 2000 (Invitrogen) with 8 µg of pCEP4-PEDF plasmid (provided by Dr Bouck, Northwestern University, Chicago, IL, USA) or control pCEP4 plasmid. Forty-eight hours later cells were selected with 300 µg/ml hygromicin B (Sigma) for two weeks, and then characterized for PEDF overexpression and used for further studies. pCEP4-PEDF plasmid contains PEDF cDNA without untranslated regions (5′UTR and 3′UTR) followed by a histidine tag at 3′.

### Plasmid Construction

Plasmids psiCHECK-3′ PEDF and psiCHECK-3′ GAPDH were generated by cloning the PEDF 3′-UTR or GAPDH 3′-UTR into the multiple cloning site of psiCHECK-2 vector (Promega) after Not I (Roche Applied Science) and Xho I (Invitrogen) digestion. The PEDF 3′-UTR and GAPDH 3′-UTR were amplified by PCR using the following pairs or oligonucleotide primers: for PEDF, primer sense 5′-cgctcgagtatcccagtttaatattcc-3′ (Xho I site underlined) and antisense 5′-cagcggccgctaacagaagttagggataa-3′ (Not I site underlined); for GAPDH, primer sense 5′-cgctcgaggaccctggaccaccagc-3′ (Xho I site underlined) and antisense 5′-cagcggccgcggttgagcacagggtac-3′ (Not I site underlined).

### UTR Reporter Assay

Reporter assays were performed using the SBcl2 and M000921 melanoma cell lines. Cells were seeded on 24-well plates (10^5^ cells/well), and 16 h later were transfected using Lipofectamine 2000 (Invitrogen) with 300 ng of empty plasmid or the indicated reported construct. Four hours after transfection, medium was changed and cells cultured for 24 h. Afterward cells were cultured under normoxic (21% O_2_) or hypoxic (1% O_2_) conditions in serum-free medium for additional 24 h. After the treatment, plates were kept frozen at −80°C until used. Analysis of Luciferase and Renilla was performed using the Dual Luciferase Reporter System (Promega) and a Lumat LB9507 luminometer (Berthold Technologies, Bad Wildbad, Germany). The Renilla activity was then normalized to Luciferase activity (constitutive expression). The results are average and standard deviation (SD) of the values obtained from two independent experiments in each melanoma cell line.

### Statistical Analysis

Statistical significance was assessed by two-tailed unpaired Student's *t-test* using GraphPad Instat (GraphPad Software, San Diego, CA, USA). *P*-values<0.05 were considered as significant.

## Supporting Information

Figure S1 **PEDF downregulation by different oxygen concentrations in melanoma cells.** Western blot analysis of extracellular PEDF (PEDF_e_) protein levels in conditioned medium (CM) and HIF1α protein levels in whole-cell extracts from M000921 melanoma cell line incubated in normoxia (21% O_2_), hypoxia (1% O_2_) and anoxia (0% O_2_) for 16 h and 24 h. β-actin was used as loading control.
(TIF)

Figure S2 **Hypoxia downregulates PEDF in melanoma cells in the absence or presence of growth factor.** (A) Western blot analysis of extracellular PEDF (PEDF_e_) protein levels in conditioned medium (CM) from WM164 melanoma cell line. (B) Western blot analysis of intracellular PEDF (PEDF_i_) and HIF1α protein levels in whole-cell extracts from WM164 melanoma cell line. Cells were grown in basal medium with or without fetal bovine serum (2% FBS or 0% FBS, respectively); or without FBS in the presence of basic fibroblast growth factor (bFGF) and epidermal growth factor (EGF) under normoxia (21% O_2_) or hypoxia (1% O_2_) for 24 h. β-tubulin was used as loading control.
(TIF)

Figure S3 **Hypoxia does not change DNA synthesis rate of primary human melanocytes and SBcl2 melanoma cells.** 5-ethynyl-2-deoxyuridine (EdU) incorporation of NHEM primary human melanocytes and SBcl2 melanoma cell grown in normoxic (21% O_2_) and hypoxic (1% O_2_) conditions. Cells were grown in normoxic or hypoxic conditions for 16 h and 24 h incubated in the presence of 20 µM EdU during the last 4 h. Bars represent average ± standard deviation (SD).
(TIF)

Figure S4 **UTRs are not required for downregulation of PEDF levels under hypoxia in M000921 melanoma.** (A) Western blot analysis of extracellular PEDF (PEDF_e_) protein levels in conditioned medium (CM), intracellular PEDF (PEDF_i_) and HIF1α protein levels in whole-cell extracts from M000921-pCEP4 and M000921-pCEP4-PEDF melanoma cell line incubated under normoxia (21% O_2_) or hypoxia (1% O_2_) for 24 h. β-tubulin was used as loading control. (B) Western blot analysis of PEDF_e_ protein levels in 24 h CM and HIF1α protein levels in whole-cell extracts from M000921 melanoma cell line incubated under hypoxia. β-tubulin was used as loading control. (C) UTR-reporter assay in M000921 melanoma cell line transfected with psiCHECK2-3′PEDF, psiCHECK2-3′GAPDH or empty vector psiCHECK2. After transfection, cells were incubated in hypoxia for 24 h. Renilla activity was normalized to luciferase activity expressed from internal control. psiCHECK2-3′GAPDH was used as a negative control. Bars represent average ± standard deviation (SD).
(TIF)

Figure S5 **Hypoxia induces the autophagic phenotype in SBcl2 melanoma cells.** Quantification of SBcl2-GFP-LC3 with autophagic vacuoles after treatment of different times of hypoxia (1% O_2_) (4 h, 8 h, 16 h, 24 h and 48 h). Ten fields from each condition were assessed. Bars represent average ± standard deviation (SD) (*P<0.05; **P<0.01).
(TIF)
