# Supplementary figures and images for: Hypoxia Negatively Regulates Antimetastatic PEDF in Melanoma Cells by a Hypoxia Inducible Factor-Independent, Autophagy Dependent Mechanism
Source: PLoS One. 2012 Mar 23;7(3):e32989. doi: 10.1371/journal.pone.0032989 (PMC3311626; doi:10.1371/journal.pone.0032989)

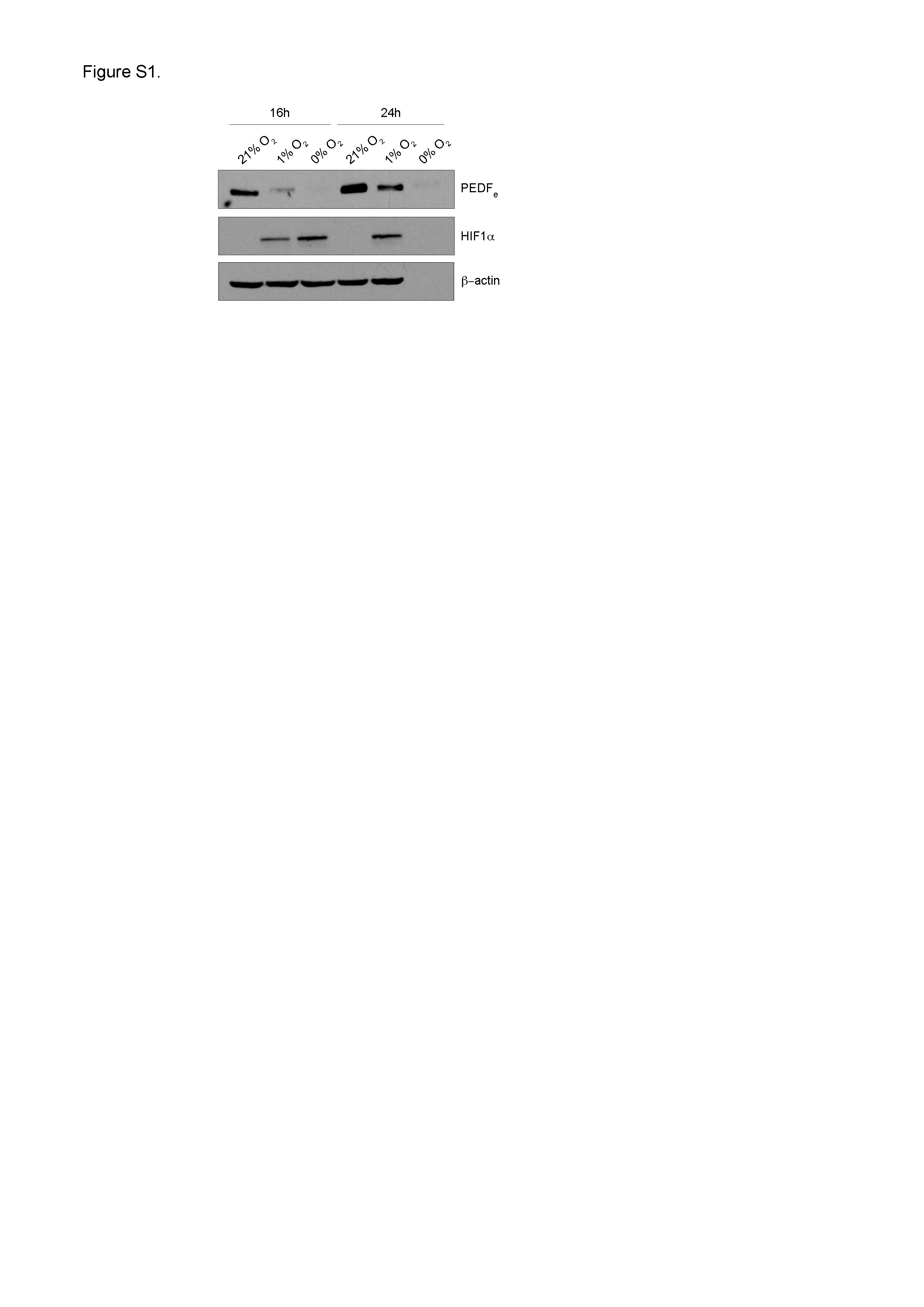

Supplement: Figure S1 — PEDF downregulation by different oxygen concentrations in melanoma cells. Western blot analysis of extracellular PEDF (PEDFe) protein levels in conditioned medium (CM) and HIF1α protein levels in whole-cell extracts from M000921 melanoma cell line incubated in normoxia (21% O2), hypoxia (1% O2) and anoxia (0% O2) for 16 h and 24 h. β-actin was used as loading control. (TIF) [file pone.0032989.s001.tif]

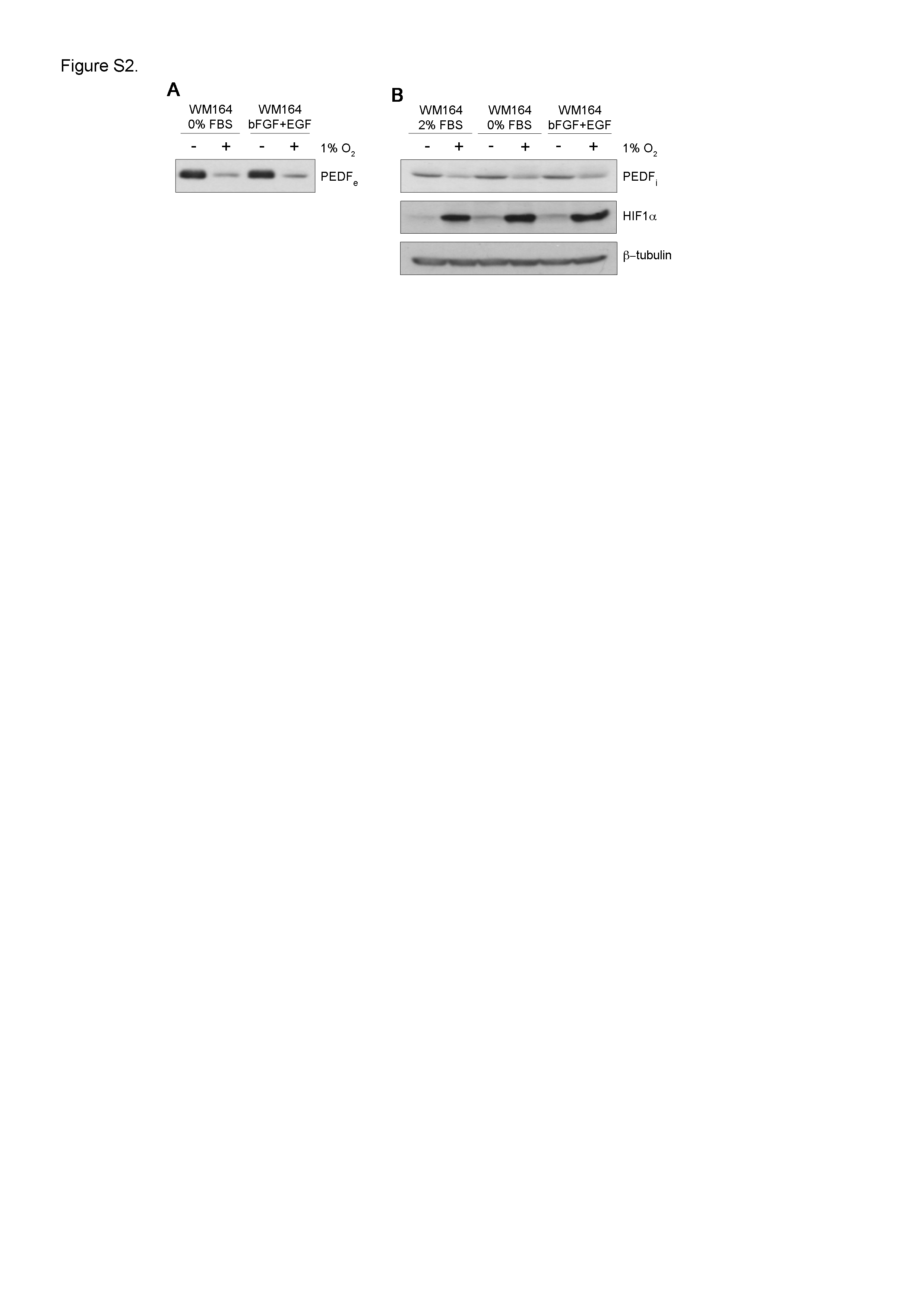

Supplement: Figure S2 — Hypoxia downregulates PEDF in melanoma cells in the absence or presence of growth factor. (A) Western blot analysis of extracellular PEDF (PEDFe) protein levels in conditioned medium (CM) from WM164 melanoma cell line. (B) Western blot analysis of intracellular PEDF (PEDFi) and HIF1α protein levels in whole-cell extracts from WM164 melanoma cell line. Cells were grown in basal medium with or without fetal bovine serum (2% FBS or 0% FBS, respectively); or without FBS in the presence of basic fibroblast growth factor (bFGF) and epidermal growth factor (EGF) under normoxia (21% O2) or hypoxia (1% O2) for 24 h. β-tubulin was used as loading control. (TIF) [file pone.0032989.s002.tif]

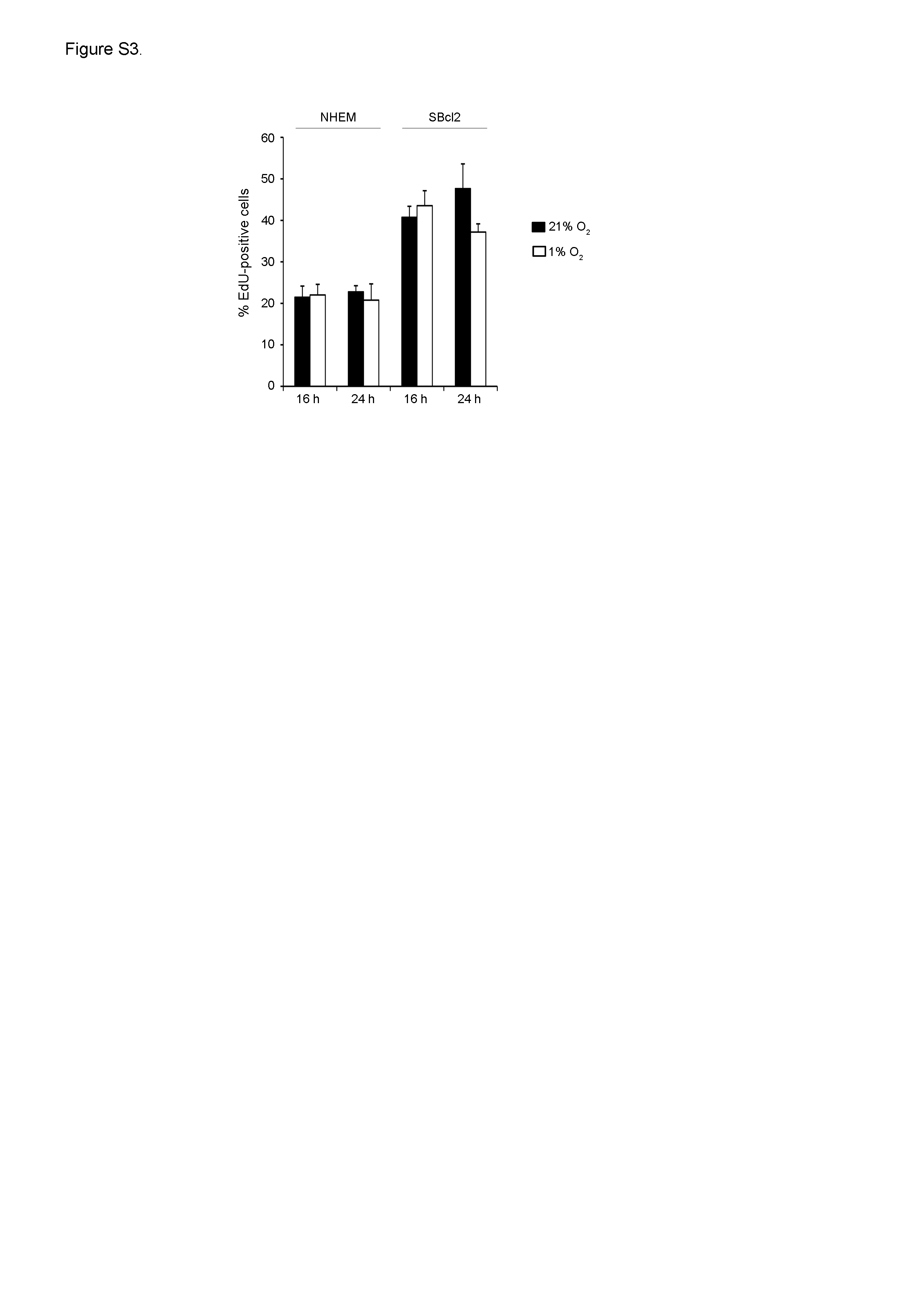

Supplement: Figure S3 — Hypoxia does not change DNA synthesis rate of primary human melanocytes and SBcl2 melanoma cells. 5-ethynyl-2-deoxyuridine (EdU) incorporation of NHEM primary human melanocytes and SBcl2 melanoma cell grown in normoxic (21% O2) and hypoxic (1% O2) conditions. Cells were grown in normoxic or hypoxic conditions for 16 h and 24 h incubated in the presence of 20 µM EdU during the last 4 h. Bars represent average ± standard deviation (SD). (TIF) [file pone.0032989.s003.tif]

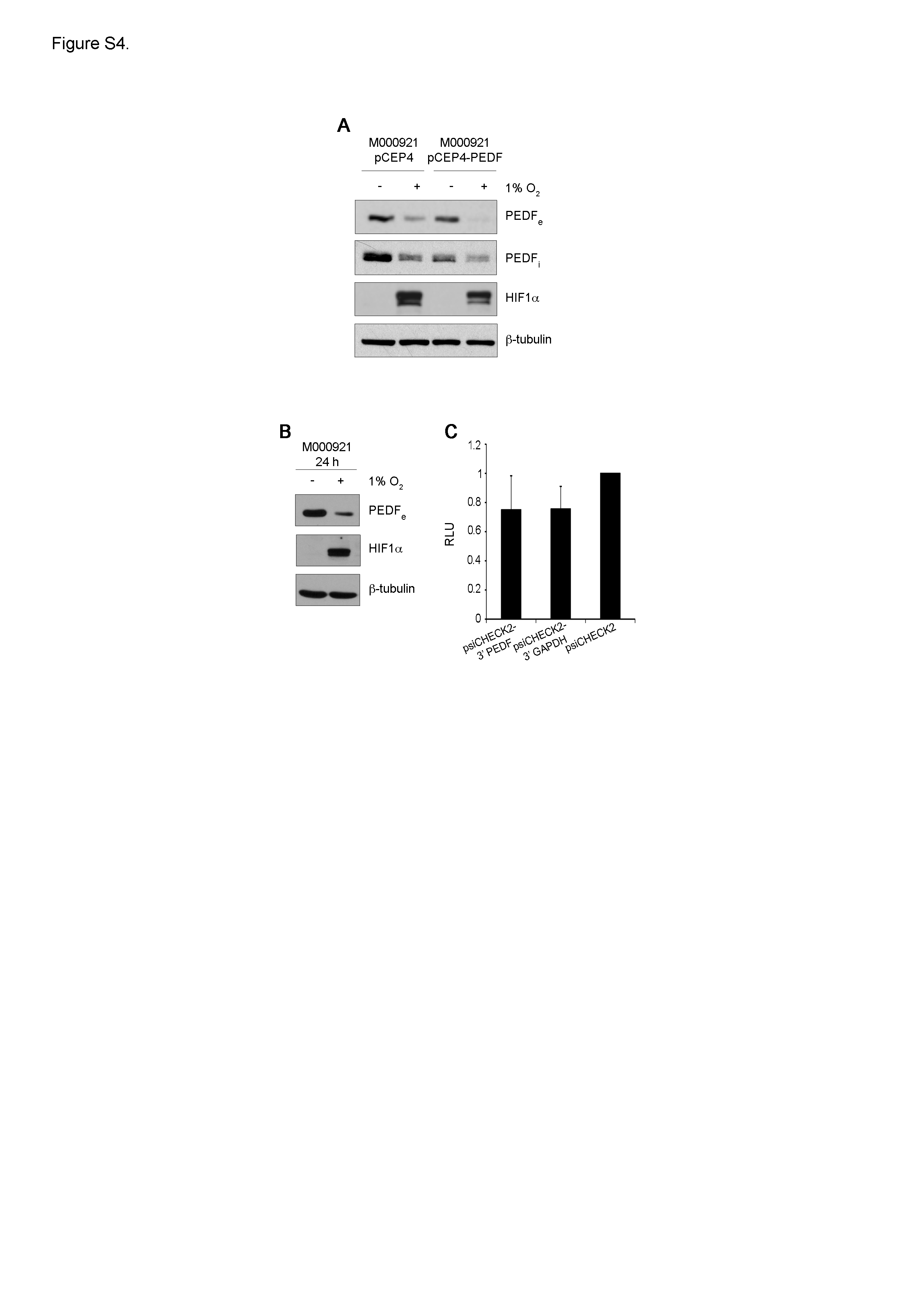

Supplement: Figure S4 — UTRs are not required for downregulation of PEDF levels under hypoxia in M000921 melanoma. (A) Western blot analysis of extracellular PEDF (PEDFe) protein levels in conditioned medium (CM), intracellular PEDF (PEDFi) and HIF1α protein levels in whole-cell extracts from M000921-pCEP4 and M000921-pCEP4-PEDF melanoma cell line incubated under normoxia (21% O2) or hypoxia (1% O2) for 24 h. β-tubulin was used as loading control. (B) Western blot analysis of PEDFe protein levels in 24 h CM and HIF1α protein levels in whole-cell extracts from M000921 melanoma cell line incubated under hypoxia. β-tubulin was used as loading control. (C) UTR-reporter assay in M000921 melanoma cell line transfected with psiCHECK2-3′PEDF, psiCHECK2-3′GAPDH or empty vector psiCHECK2. After transfection, cells were incubated in hypoxia for 24 h. Renilla activity was normalized to luciferase activity expressed from internal control. psiCHECK2-3′GAPDH was used as a negative control. Bars represent average ± standard deviation (SD). (TIF) [file pone.0032989.s004.tif]

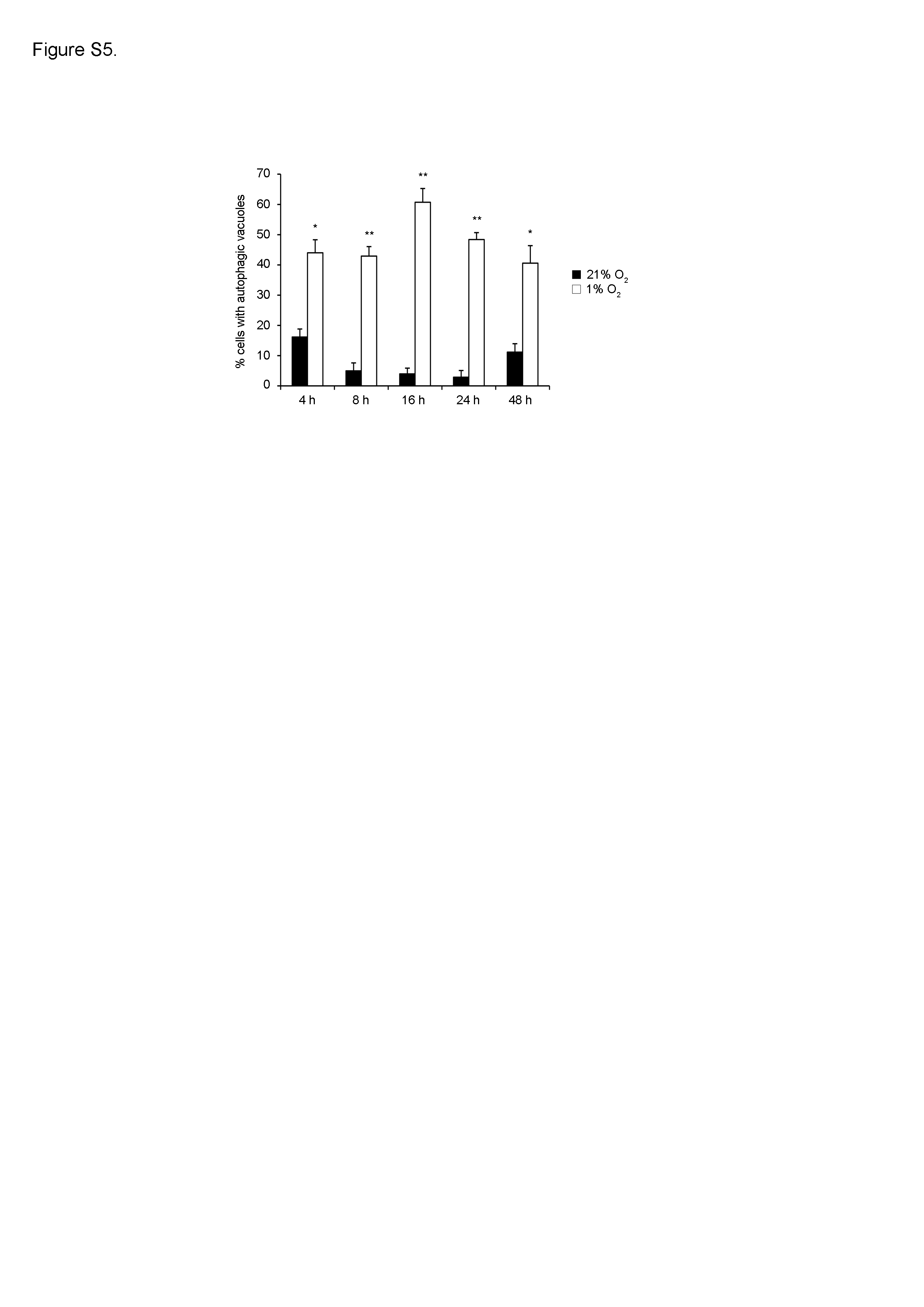

Supplement: Figure S5 — Hypoxia induces the autophagic phenotype in SBcl2 melanoma cells. Quantification of SBcl2-GFP-LC3 with autophagic vacuoles after treatment of different times of hypoxia (1% O2) (4 h, 8 h, 16 h, 24 h and 48 h). Ten fields from each condition were assessed. Bars represent average ± standard deviation (SD) (*P<0.05; **P<0.01). (TIF) [file pone.0032989.s005.tif]
